# An interactive web-based intervention on nutritional status, physical activity and health-related quality of life in patient with metabolic syndrome: a randomized-controlled trial (The Red Ruby Study)

**DOI:** 10.1038/nutd.2016.35

**Published:** 2017-01-09

**Authors:** L Jahangiry, A Montazeri, M Najafi, M Yaseri, M A Farhangi

**Affiliations:** 1 Health Education and Health Promotion Department, School of Public Health, Tabriz University of Medical Sciences, Tabriz, Iran; 2 National Public Health Management Center, Tabriz University of Medical Sciences, Tabriz, Iran; 3 Mental Health Research Group, Health Metrics Research Center, Iranian Institutes for Health Sciences Research, ACECR, Tehran, Iran; 4 Tehran Heart Center, Tehran University of Medical Sciences, Tehran, Iran; 5 Department of Epidemiology and Biostatistics, School of Public Health, Tehran University of Medical Sciences, Tehran, Iran; 6 Nutrition Research Center, Department of Community Nutrition, Faculty of Health and Nutrition, Tabriz University of Medical Sciences, Tabriz, Iran

## Abstract

**Background/Objectives:**

Physical inactivity and unhealthy nutritional behaviors are recognized as the key factors in the cause and management of metabolic syndrome (MetS). The effectiveness of interactive web-based interventions on dietary intakes, physical activity and health-related quality of life (HRQOL) among people with MetS is currently unknown.

**Methods:**

The two-arm randomized-controlled trial was conducted for patients with MetS from June through December 2012 in Tehran, Iran. Participants (*n=*160) were recruited through online registration on the study website. After free clinical assessments of eligible participants for MetS, they were randomly assigned to intervention and control groups (*n=*80). All participants received general information about cardiovascular diseases and MetS risk factors via the website. The intervention group logged in to interactive part including My Healthy Heart Profile, received tailored calorie-restricted diet and used all parts of the interactive prevention program. Anthropometric measures, glycemic status, lipid profile, physical activity and food intake were evaluated at the beginning and after 6-month follow-up. HRQOL was assessed at beginning, 3- and 6-month follow-up.

**Results:**

There were no significant differences between the intervention and control groups on age, gender, education and MetS factors. In comparison with control group, the intervention group showed significant changes in moderate physical activity 260.3±473.6 vs 101.6±213.1 MET-min/week, walking 505.2±505.3 vs 321±884 MET-min per week, cholesterol intake −88.4±158.7 vs −8.3±6 mg per day, total calories −430.2±957.5 vs −392.9±34.7 kcal per day and sodium 1336.9±2467 vs 1342±3200.4 mmol per day. With regard to HRQOL, the intervention group showed greater improvement in general health and vitality (*P<*0.05 for all).

**Conclusion:**

These results indicate the positive impact of a lifestyle intervention by a web-based program on physical activity, dietary intake and several dimension of QoL. The use of web-based approaches is a great interest to manage patients at high cardiovascular risk, especially where the prevalence of obesity, MetS and diabetes is increasing.

## Introduction

Metabolic syndrome (MetS) is becoming a worldwide epidemic disorder that increases the risk of cardiovascular diseases.^1^ The disorder is defined as a cluster of most dangerous heart attack risk factors including abdominal obesity, increased triglycerides, reduced high-density lipoprotein cholesterol (HDL), hypertension and glucose intolerance.^2^ The prevalence of MetS has been increasing worldwide.^3^ Iran has one of the highest rates of the prevalence of the MetS. Recent evidence from Lipid and Glucose study among adult population in Tehran indicated that MetS appears to affect ~34.7% of Iranian population.^4, 5^ Third report of the National Cholesterol Education Program (NCEP) adult treatment panel (ATP III) declared that the presence of the MetS was identified by three or more of the following components: waist circumference >102 cm in men and >88 cm in women (for Iranian, >90 cm for both genders^6, 7^), triglyceride level of at least 150 mg dl^−1^, HDL level <40 mg dl^−1^ in men and <50 mg dl^−1^ in women, systolic/diastolic blood pressure 130/85 mmHg or higher and fasting blood glucose level 110 mg dl^−1^ or higher.^8^ Lifestyle modification strategies are foremost in the management of MetS.^9^ Lifestyle interventions that mainly consist of increasing physical activity and improving dietary habits have been demonstrated to improve MetS. Lifestyle programs with increased physical activity and focused on healthy dietary help people for weight loss, raises HDL cholesterol, lowers serum cholesterol, triglycerides, glucose and blood pressure.^10, 11^

Current evidence indicates that poor health-related quality of life (HRQOL) was associated with the number of MetS components.^12^ Several components of MetS such as abdominal obesity, hypertension and diabetes have been associated with lower HRQOL.^13, 14^ In line with this evidence Ford and colleagues indicated that of five components of MetS abdominal obesity and hyper trigliceridemia showed inverse association with HRQOL.^15^

Previous studies have shown that web-based interventions could provide a solution for lifestyle modification and quality of life.^16, 17, 18, 19^ No study has examined the effects of interactive interventions on MetS. The aim of this study was to evaluate the effectiveness of an interactive web-based intervention on nutritional status, physical activity and HRQOL among people with MetS.

## Material and methods

### Design

**Figure 1:**
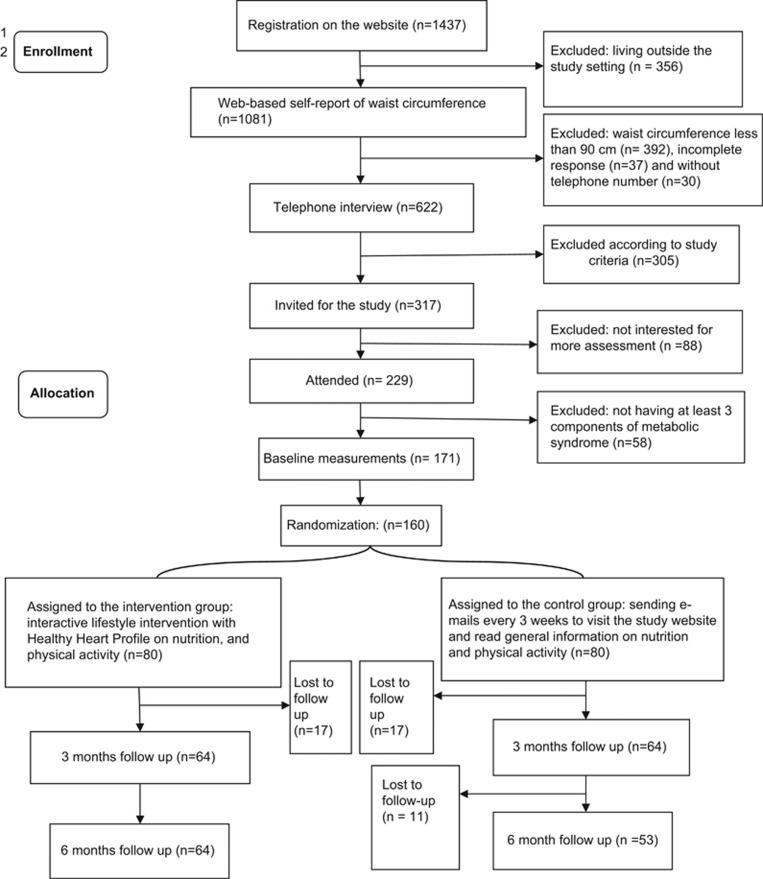
The study flowchart at a glance.

Figure 1 shows the study process. The study was a part of a bigger research project on the evaluation of an interactive web-based lifestyle program on MetS. This was a 6-month randomized-controlled trial (IRCT201111198132N1) that conducted during Jun to December 2012 in Tehran Heart Center, Tehran, Iran. The methodological details has been documented previously in the other study.^20^

### Participants, recruitment and randomization

Participants with MetS were assessed at two points in time: at baseline and 6 months follow-up. Recruitment was begun through an advertisement that placed in the virtual and non-virtual environments including Tehran Heart Center, Tehran, Iran. The advertisement introduced the web-based lifestyle study (http://www.Heartresearch.ir). Participants who visit the study website were submit the information required for registration as name, gender, waist circumference, weight e-mail and address by registration link on the study website. Then, in reviewing registration database, participants who have aged 20 years and above and living in Tehran were contacted by a telephone call and screened for eligibility. Finally, the eligible interested participants were invited to free clinical assessment by a trained research assistant at Tehran Heart Center. Participants Randomization performed after baseline measurements and randomly assigned to the intervention and control arms by sequencing participants assignments by block size of 4.

The inclusion criteria were: (a) waist circumference⩾90 (cutoff for MetS in Iran for both gender^6, 7, 21^), (b) blood pressure⩾130/85, (c) access to Internet, (d) having at least three components of MetS, (e) having simple skills to work with Internet. Exclusion criteria included: (a) having history of cardiovascular diseases, (b) diabetics, (c) having cancer, (d) patients with renal diseases, (e) being pregnant, (f) taking medication for hypertension, (g) taking medication for dislipidemia and (h) having incomplete registration form.

### Intervention and control

All study participants in intervention and control groups received general information about cardiovascular diseases and MetS indicators through the website and e-mail. The information about healthy nutrition and physical activity were sent for each participant. Also, they were informed of their high-risk conditions by identifying number of MetS components. The interactive parts named My Healthy Heart Profile were used by intervention group through study website. They received username and password to use the personal page. My Healthy Heart Profile was a free interactive web-based program that developed for prevention of cardiovascular diseases. The intervention program was developed according to the ‘feedback and monitoring' as a behavior change technique.^22^ It was designed to send feedback to each participant according to his/her MetS indicators. In fact the participants received text-based messages and graphic feedbacks immediately after compellation of each record of risk factors. The program in five parts including: (1) personal homepage (containing same educational materials in titles; Do you know your cholesterol level?, Control your high blood pressure, Are you at risk for heart disease, Do you need to lose weight?, Protect your heart against diabetes, Physical activity and your heart, and Lowering blood pressure. The homepage updated at least twice a month. (2) Personal information: including recording of name, age, gender, weight, height, telephone number and e-mail addresses. (3) Message inbox was the section for reciprocal communications between administers and users. At beginning of the intervention a dietician (MAF) was provided the calorie-restricted tailored diet^23^ to participants and send to their message inbox. An e-mail as a reminder had been sent to participants that notified a new message or updated materials of the My Healthy Heart Profile. Users could ask their answers at any time they wished and received their response within 24 h. (4) Interactive Framingham stroke risk estimated cardiovascular risk for 10 years (based on the Persian online version of Framingham risk score by permission). The Framingham stroke risk considers six cardiovascular risk factors, including age (over than 20), gender (male and female), total cholesterol, HDL cholesterol, systolic blood pressure and smoking habits. Participants could estimate their scores in every log-in and obtained feedback via the text by the three traffic lights that were illustrated for three levels of risks (high, moderate and low risk). (5) Measurements were another section for periodic records of anthropometric and clinical assessments including: weight, waist circumference, body mass index (BMI) and blood pressure, Total cholesterol, LDL-cholesterol, HDL cholesterol, triglycerides and fasting blood glucose. A simple graph were displayed the measurements by three warning color (red=needs attention, orange=close to risk and green=good) for each recording. Participants in the control group as a waiting list received e-mail messages every 3-weeks that included information about MetS and general information about healthy nutrition and benefits of fruit and vegetable intakes, physical activity and body weight loss.

### Outcome measures

#### Cardio-metabolic risk factors

MetS status was determined using the National Cholesterol Education Program (NCEP) Adult Treatment Panel (ATP) III criteria.^24^ The primary outcomes were the change in weight, BMI, abdominal obesity, elevated systolic/diastolic blood pressures, impaired fasting glucose, elevated triglycerides and Low HDL cholesterol. A measuring tape to the nearest of 0.1 cm were used for evaluating waist circumference and measured in horizontal plane, midway between the lowest rib and the iliac crest with a measuring tape in centimeter.^25^ The weight of individual dressed in light clothing without shoes were measured by using a calibrated scale (Seca, Hamburg, Germany model 8811021658) to the nearest of 0.1 kg. Height was measured without shoes using a stadiometer (Seca, Hamburg, Germany) to the nearest of 0.1 cm. BMI was measured by using the individual's weight in kilograms divided by the square of his/her height in meters.^26^ Blood pressure was measured with mercury sphygmomanometer twice in the same arm after the individual seated at rest 10–15 min. The systolic and diastolic measurement were represented the mean of two readings. Blood sampling was collected for measurements of total cholesterol, triglycerides, LDL-cholesterol, HDL cholesterol, fasting blood glucose for all participants included in the study. Overnight fasting for 12–14 h were needed before blood sampling. Fasting blood glucose was measured using the glucose oxidase method (intra and inter-assay coefficients of variation of 2.1% and 2.6%, respectively).

#### Physical activity and nutritional status

Physical activity was measured using the International Physical Activity Questionnaire at last 7 days (IPAQ) that was well-validated questionnaire in Iran.^27^ The validated Iranian version of Food Frequency Questionnaire (FFQ) was used for food frequency measurement.^28^

#### HRQOL

HRQOL was measured with Short Form-36. The SF-36 is a generic measure designed to evaluate self-reported health status, functioning and well-being. The psychometric properties of the Iranian version of SF-36 are well documented.^29^

### Sample size

The sample size was calculated based on one s.d. decrease (2.5 cm)^30^ in waist circumference as the primary outcome measure. As such a study with a power of 90 at 5% significance level would need 60 participants in each arm. Giving that there might be an attrition risk, 80 participants per each group were sought.

### Statistical analysis

To present data we used mean, s.d., frequency and percent. Normal distribution of data were assessed by Kolmogrov–Smirnov test and Q–Q plot. To compare the variables in the baseline between two groups, we used *t*-test, Mann–Whitney test, *χ*^2^ and Fisher's exact test. To evaluate the changes within groups for HRQOL, we used mixed model analysis. Adjusted for the multiple comparison was done by Bonferroni method. Another Mixed model was used to compare the trend of changes between two groups. All comparison between treatment groups was based on intention-to-treat analysis using multiple imputation method.

All statistical analysis performed by SPSS (Version 21.0, IBM Co., Chicago, IL, USA). *P*-values <0.05 considered statistically significant.

### Ethics

The procedures followed were in accordance with the ethics standards of the Tehran University of Medical sciences Ethics Research Committee. We obtained written informed consent from all participants.

## Results

### General characteristics, physical activity and nutritional status

The study as a part of larger project presents secondary outcomes of the results. It should be noted that the primary outcomes have been published elsewhere.^31^ As reported, earlier distribution of age, gender, education and cardio-metabolic risk factors do not show any significant differences between two groups. The mean age of participants was 44.2 (s.d.=10.0) years. The mean weight and BMI were 87 kg (s.d.=15) and 30.1 kg m^−2^ (s.d.=4.6), respectively.

Participants were predominantly male (66.3%) and well educated (55.6% with >12 years education). The characteristics associated with the MetS among the participants are summarized in Table 1 that shows intervention and control groups were similar at baseline for all variables. The prevalence of MetS and its components were decreased significantly in both intervention and control groups during the intervention. Significant within group differences were found for weight (*P<*0.001), BMI (*P<*0.001), abdominal obesity (*P<*0.001), elevated systolic blood pressure(*P<*0.001), elevated diastolic blood pressure (*P<*0.001), impaired fasting glucose (*P=*0.046), elevated triglycerides(*P<*0.001) in intervention group and for weight (*P<*0.003), BMI (*P<*0.003), elevated systolic blood pressure (*P<*0.001), elevated diastolic blood pressure (*P<*0.001), impaired fasting glucose(*P<*0.001), elevated triglycerides(*P<*0.001) in control group. From the baseline to the end of the study, a significant between-group differences were observed for elevated diastolic blood pressure (*P=*0.013), impaired fasting glucose (*P=* 0.012) and elevated triglycerides (*P=*0.004).

Physical activity, dietary energy and nutrient intake were presented in Table 2. Comparison of Physical activity and dietary data between intervention and control groups showed no significant differences in the initial values. The significant changes were found between intervention and control groups for moderate physical activity (260.3±473.6 vs 101.6±213.1) and walking (505.2±505.3 vs 321±884), respectively, (*P<*0.05). Comparison of the dietary variables showed significant decrease in both intervention and control groups for total fat (–19.7±73.2 vs –15.2±57.1) and sodium intake (1336.9±2467 vs 1342±3200.4) and significant changes for total calories (–430.2±957.5 vs –392.9±34.7) and cholesterol (88.4±158.7 vs 8.3±6), respectively, between intervention and control groups. Also within group differences remained significant for polyunsaturated fat (7.3±15.8 vs −2.8±10.4), carbohydrate (−67.6±153.2 vs −51.2±10.2) and fiber (5.7±23 vs −9.7±39.9), respectively, between intervention and control groups.

The comparison of daily intake of food groups showed that there were no statistically significant differences between intervention and control groups at baseline (Table 3). The significant reduction were observed for meat/eggs from 133.1±104.6 to 88.7±70.7 (P=0.033), fat/oils from 22.5±18.7 to 15.1±8.8 (*P=*0.049) and others from 909.9±490.3 to 808±695.4 (*P=*0.033) in the intervention group after 6-month intervention. Also, vegetable consumption was increased significantly from 374.4±213.6 to 395.7.

In comparison of attrition rate in the intervention and control groups, the control group had significantly higher attrition rate (%33.7) than the intervention group (%20) at 6 months follow-up. The more information of the topic was report previously in details.^32^

### HRQOL

The results of the analysis of HRQOL based on mixed model, adjusted for the multiple comparisons by Bonferroni method are shown in Table 4. There were significant interactions between groups over time for general health (10±18.7, 18.1±18.1) and vitality (5.1±15.9, 10.2±13.8) at 3- and 6-month, respectively, (*P<*0.05). The comparison of change within group showed significant differences for physical function (4.9±9.8 versus 3.4±12.4), bodily pain (16.1±17.9 versus 3.1±16.1), general health (18.1±18.1 versus 8.2±17.8), vitality (10.2±13.8 versus 1.8±13.9), role emotional (14.4±46.1 versus 6.8±33.7) and mental health (4.2±14.8 versus −0.4±16.6), respectively, in intervention and control group at 6 month. The pattern of change showed that some subscales of HRQOL improved significantly in both intervention and control group such as bodily pain, general health and vitality at 3 month.

### The usage of intervention program

The usage of intervention program was evaluated by log-ins frequency to my healthy heart profile. The mean number and standard error of log-ins to the intervention program at 6 months were 5.00. The mean (s.e.) of log-ins to the program at the periods was 6.01 (0.579). Participants who logged in five or more times during the intervention period treated significantly MetS (Fishers's exact test 8.36, *P*=0.004).

## Discussion

This study assessed the interactive web-based intervention effects on patients with MetS to improve physical activity; dietary intake and HRQOL. The 6-month web-based intervention was resulted in significant changes in physical activity and improvement in dietary intakes (*P<*0.05).

This study showed that interactive web-based program with feedback and monitoring produced significant relatively improvement in adopting a healthy diet and physical activity habits, these can lead to more improvement in MetS indicators over time. The systematic reviews of randomized-controlled trials have concluded that lifestyle interventions can be effectively delivered over the Internet.^33, 34^ The previous study has been indicated that there is an association between specific characteristics of Internet interventions and better exposure to the intervention and its contents.^35^ So, the number of interactive elements was resulted in more log-ins to the intervention website.^36, 37^ To obtain an objective measure of website use, log-in data for intervention groups were tracked over 6 month. Participants in the intervention group logged in to the website a mean (s.e.) of 6.01 (0.579). Participants who logged in more than five time to intervention website at 6 months and use of interactive elements of the website including, self-monitoring of MetS indicators (such as waist circumference, HDL cholesterol, fasting blood glucose, triglycerides, blood pressures), weight, BMI and received the feedback tailored with their conditions was related to successful improvement in lifestyle related to MetS. Majority of studies suggest that web-based interventions with some features including self-monitoring, feedback/tailored information have a beneficial effect on behavior change.^38, 39^ Vandelanotte *et al.* reported in a review that better outcome measures regarding improvement of physical activity were identified when participants visited the intervention website more than five times.^40^ McManus *et al.* in a study on self-monitoring of blood pressure reported that the majority of participants have used appropriate schedules of measurements.^41^ Another behavioral weight loss studies revealed significant detail about self-monitoring patterns to improve the adherence to the interventions.^42, 43^

The goal behind the design and development of the Healthy^44^ Heart Profile was to create a profiling and individualized management of cardiovascular diseases risk factors via the web to monitor and assess the risks factors efficiently and effectively than the alternatives. Recent study presents a web-based cardiovascular risk assessment with tailored feedback and linkage to health management and lifestyle providers proved the feasibility and effectiveness of the program. Similar to our study this study showed that for eliciting actual behavior change, the personalized prevention approach may offer a system for integrated risk profiling and helped to the health risk assessment.^39^

Owing to the public health importance of MetS, lifestyle interventions must be effective and available and accessible to the high-risk populations. The Internet and technology programs provide unique opportunity for developing and implementing of lifestyle interventions that promote feedback and monitoring.^45, 46^

Although analyses revealed significant improvements over time in physical activity and dietary behaviors in both groups, but intervention group showed relatively greater improvements in some physical activity and dietary behaviors. Similar to our study, Tate *et al.*^37^ showed that delivering a structured behavioral treatment program with weekly contact and individualized feedback had better weight loss and showed greater reductions in waist circumference compared with those given links to educational websites. Total caloric intake and cholesterol were significantly reduced by our lifestyle program. Also within group significant modification were obtained in fiber, carbohydrate, polyunsaturated fat and sodium. These reductions are similar to those reported in previous studies.^11, 27^

Another major finding of the study was the beneficial effect of intervention on HRQOL especially for general health and vitality after adjusted for the multiple comparisons. Also, the positive results were observed within groups for physical function, bodily pain, general health, vitality, role emotional and mental health in both intervention and control groups. A large body of research found that people with MetS report lower HRQOL than people without MetS.^12, 14, 47^ Our results were consistent with other studies.^48, 49^ Oh *et al.*^48^ reported that therapeutic lifestyle modification had been resulted in greater improvements in physical function (*P*=0.017), general health (*P*=0.001), vitality (*P*=0.008) and mental health (*P*=0.027). These findings suggested that lifestyle intervention could effectively improve HRQOL in population with MetS. The results of a 12-week web-based randomized-controlled trial suggest that the Internet intervention with tailored physical activity counseling can have beneficial short-term effect on cardiorespiratory fitness, HRQoL and BMI among adolescents with overweight and obesity.^50^

### Limitations

This study was not able to assess the frequency of website use in control groups. In this study, participants were predominately well educated and male. This may limit the generalizability of our results.

## Conclusion

Our results give preliminary supports to the effectiveness of an interactive web-based intervention to the improving nutritional status, physical activity and HRQOL in patient with MetS. The integration of interactive e-health programs to primary health care practices such as prevention of cardiovascular risk factors offers possibilities for on- time interaction with the target group with several advantages for the users as well as for the administers of the preventive programs. The use of web-based approaches is a great interest in the management of patients at high cardiovascular risk, especially in a scenario where the prevalence of obesity, MetS and diabetes is increasing. There is a need to future interventions applying newer technology in prevention of cardiovascular diseases.

## Figures and Tables

**Table 1:** Comparison of baseline and final values of the metabolic syndrome components in the two groups

	*Intervention*			*Control*			*P^ii^*	*P^iii^*
	*Baseline (*n=*80)*	*After 6 month (*n=*63)*	*P^i^*	*Baseline (*n=*80)*	*After 6 month (*n=*53)*	*P^i^*		
Weight (Kg) (mean, s.d.)	87±16	83±15	<0.001	88±14	87±12	0.003	0.626	0.046^a^
Body mass index (Kg m^−2^) (mean, s.d.)	29.8±4.7	28.6±4.4	<0.001	30.5±4.5	29.5±3.5	0.003	0.354	0.195^a^
Abdominal obesity (number, %)	103 (8.9)	99.1 (9)	<0.001	105 (8.4)	100.1 (17.5)	0.073	0.999	0.265^b^
Elevated systolic blood pressure (number, %)	63 (78.7)	18 (28.5)	<0.001	62 (77.5)	19 (35.8)	<0.001	0.848	0.479^b^
Elevated diastolic blood pressure (number, %)	71 (88.7)	13 (20.6)	<0.001	68 (85)	22 (41.5)	<0.001	0.482	0.013^b^
Impaired fasting glucose (number, %)	6 (7.5)	0 (0)	0.046	8 (10)	5 (9.4)	<0.001	0.576	0.012^c^
Elevated triglycerides (number, %)	62 (77.5)	6 (9.5)	<0.001	63 (78.7)	16 (30.2)	<0.001	0.848	0.004^c^
Low HDL cholesterol (number, %)	61 (76.2)	10 (15.8)	0.001	53 (66.2)	14 (26.4)	0.655	0.751	0.150^b^

Abbreviations: HDL, high-density lipoprotein; P^i^, within group difference; P^ii^, between group at the baseline; P^iii^, between group after the intervention.

a Derived from *χ*^2^-test.

b Derived from *χ*^2^-test.

c Derived from Fisher's exact test.

**Table 2:** Physical activity, dietary energy and nutrient intake among participants

	*Intervention (*n=*80)*			*Control (*n=*80)*			*P*^*ii*^	*P*^*iii*^	*P*^*c*^
	*Baseline (mean, s.d.)*	*Change (mean, s.d.)*	*P^i^*	*Baseline (mean, s.d.)*	*Change (mean, s.d.)*	*P^i^*			
*Physical activity MET-min per week*									
Vigorous	111.7 (291.4)	133.3 (545.1)	0.173	73.7 (319.5)	209.4 (580)	<0.001	0.463	0.545	0.245
Moderate	41.2 (88.2)	260.3 (473.6)	<0.001	43.1 (119.6)	101.6 (213.1)	0.059	0.050	0.046	0.006
Walking	300.6 (418.7)	505.2 (505.3)	<0.001	406.3 (0.625)	321 (884)	0.247	0.585	0.045	0.048
Total metabolic equivalent of activity	453.2 (724)	898.3 (1222)	<0.001	522.6 (609.5)	580.3 (1233.7)	0.001	0.576	0.156	0.035
									
*Nutrition intake*									
Total calories (kcal per day)	2943.4 (1227.3)	−430.2 (957.5)	0.003	3036.6 (996.3)	−392.9 (34.7)	0.058	0.436	0.034	0.105
Total fat (mg per day)	95.1 (51.5)	−19.7 (73.2)	0.028	95.6 (38.7)	−15.2 (57.1)	0.037	0.839	0.563	0.71
Cholesterol (mg per day)	285.6 (147.6)	−88.4 (158.7)	<0.001	276.6 (153.2)	−8.3 (60)	0.878	0.876	0.007	0.021
Saturated fat (g per day)	25.9 (12.9)	0.33 (30.3))	0.94	26.8 (14)	−0.90 (23.4)	0.25	0.780	0.878	0.443
Polyunsaturated fat (g per day)	14.3 (4.7)	7.3 (15.8)	0.046	18.8 (9.8)	−2.8 (10.4)	0.068	0.049	0.111	0.583
Protein (g per day)	108.4 (44.2)	−34.2 (28.2)	0.75	103.3(44.1)	−8.30 (21.7)	0.096	0.414	0.44	0.37
Carbohydrate (g per day)	459.7 (189.4)	−67.6 (153.2)	0.004	475.2 (160.3)	−51.2 (10.2)	0.137	0.769	0.037	0.065
Fiber (g per day)	45.9 (19.5)	5.7 (23)	0.015	60.6 (34.4)	−9.7 (39.9)	0.097	0.225	0.189	0.543
Sodium (mmol per day)	4196.1 (1828.5)	1336.9 (2467)	0.007	4379.7 (2509.1)	1342 (3200.4)	0.006	0.280	0.179	0.049

Abbreviations: P^i^, within group difference; P^ii^, between group at the baseline; P^iii^, between group after the intervention; P^c^, change between group;

MET, metabolic equivalent. Derived from *t*-test.

**Table 3:** Daily intakes of the food groups

	*Intervention*			*Control*			*P*^*ii*^	*P*^*iii*^
	*Baseline (mean, s.d.)*	*After (mean, s.d.)*	*P^i^*	*Baseline (mean, s.d.)*	*After (mean, s.d.)*	*P^i^*		
Grains/cereals (g per day)	492.7±223/2	383.9±165.3	0.001	558.5.±286.7	465.9±382.4	0.004	0.113	0.332
Meat/eggs	133.1±104.6	88.7±70.7	0.004	122.5±78.7	118.4±	0.234	0.276	0.033
Dairy products	402.3±260.9	415.4±213.6	0.710	456.8±361.3	421.8±319.4	0.807	0.283	0.410
Fruits	885.4±760.5	895±760.5	0.945	933.8±674.2	974±649.8	0.395	0.659	0.582
Vegetables	374.4±213.6	395.7±184.6	0.726	366.9±181.3	368.3±171.2	0.569	0.776	0.044
Lentils/legumes	46.1±48.8	51.2±46.3	0.746	48.1±45.1	48.1±27.6	0.167	0.859	0.050
Seeds	17.2±64.7	20.3±14.4	0.355	18.5±47.7	20.2±67.1	0.451	0.883	0.27
Fat/oils	22.5±18.7	15.1±8.8	0.042	21.6±18.8	19.3±15.9	0.32	0.98	0.049ed
Others	909.9±490.3	808±695.4	0.05	931.3±727.7	974±649.6	0.988	0.814	0.033

Abbreviations: P^i^, within group difference; P^ii^, between group at the baseline; P^iii^, between group after the intervention.

Derived from *t*-test.

**Table 4:** Changes in health-related quality of life over time between the two groups

	*Time*	*Control*	*Intervention*	*Time group interaction*^a^
Physical function	Baseline			
	Value	89.6±15.9	93.3±11.1	0.935
	Month 3			
	Value	92.1±13	95.2±11.9	
	Change	1±9.8	2±14.8	
	*P*-value^b^	0.77	0.385	
	Month 6			
	Value	94.6±10	98.1±4.9	
	Change	3.4±12.4	4.9±9.8	
	*P*-value^b^	0.061	0.007	
Role physical	Baseline			
	Value	81.1±34.9	85.8±30.1	0.629
	Month 3			
	Value	83.2±29.6	91.5±24.7	
	Change	1.6±20.6	6.1±28.8	
	*P*-value^b^	>0.99	0.13	
	Month 6			
	Value	90.4±21.7	91.5±21.2	
	Change	7.8±30.4	6.1±25.6	
	*P*-value^b^	0.096	0.267	
Bodily pain	Baseline			
	Value	49.2±28.4	52.5±26.5	0.161
	Month 3			
	Value	39.5±27.9	78.9±25	
	Change	−15.8±26.9	26.3±34.2	
	*P*-value^b^	0.002	<0.001	
	Month 6			
	Value	58.8±27.4	68.8±26.7	
	Change	3.1±16.1	16.1±17.9	
	*P*-value^b^	0.147	<0.001	
General Health	Baseline			
	Value	60.2±16.6	58.5±16.2	0.01
	Month 3			
	Value	61.7±15.3	68.6±19.8	
	Change	–0.5±8.6	10±18.7	
	*P*-value^b^	<0.001	<0.001	
	Month 6			
	Value	69.9±16.5	76.7±13	
	Change	8.2±17.8	18.1±18.1	
		>0.99	<0.001	
Vitality	Baseline			
	Value	58.6±18.3	59.3±15.5	0.033
	Month 3			
	Value	65.2±15.9	64.8±15.6	
	Change	3.5±8.7	5.1±15.9	
	*P*-value^b^	0.031	0.01	
	Month 6			
	Value	63.3±14.3	69.8±13.2	
	Change	1.8±13.9	10.2±13.8	
	*P*-value^b^	0.3	<0.001	
Social functioning	Baseline			
	Value	83.2±25.9	87.1±20	0.865
	Month 3			
	Value	87.9±19	91.1±16.4	
	Change	2.1±11	3.8±20.5	
	*P*-value^b^	0.435	0.167	
	Month 6			
	Value	92.7±15.1	95±10.9	
	Change	6.6±22.6	7.4±16.4	
	*P*-value^b^	0.011	0.007	
Role emotional	Baseline			
	Value	70.1±41.1	71.3±40.3	0.635
	Month 3			
	Value	70.1±36.9	80.1±31.9	
	Change	−3.2±21.6	9.7±44.4	
	*P*-value^b^	>0.99	0.106	
	Month 6			
	Value	82.7±32.2	85.3±28.9	
	Change	6.8±33.7	14.4±46.1	
	*P*-value^b^	0.114	0.021	
Mental health	Baseline			
	Value	64.1±18.4	65.3±15.6	0.269
	Month 3			
	Value	68.3±16.8	68.8±14.2	
	Change	0.9±5.4	2.7±14.8	
	*P*-value^b^	0.595	0.156	
	Month 6			
	Value	66.6±15.1	70.4±13.6	
	Change	–0.4±16.6	4.2±14.8	
	*P*-value^b^	>0.99	0.046	
Physical health	Baseline			
	Value	70.1±17.4	73.3±13.6	0.884
	Month 3			
	Value	75.3±16.6	80±12.6	
	Change	1.2±6.9	6.5±12.1	
	*P*-value^b^	0.428	<0.001	
	Month 6			
	Value	82.6±10.3	83.6±10.4	
	Change	8.1±16.2	9.3±11.9	
	*P*-value^b^	<0.001	<0.001	

a Based on mixed model.

b Based on mixed model, adjusted for the multiple comparisons by Bonferroni method.
